# Mutagenicity of Flavonoids Assayed by Bacterial Reverse Mutation (Ames) Test

**DOI:** 10.3390/molecules17055255

**Published:** 2012-05-07

**Authors:** Flavia Aparecida Resende, Wagner Vilegas, Lourdes Campaner dos Santos, Eliana Aparecida Varanda

**Affiliations:** 1 Department of Biological Sciences, Faculty of Pharmaceutical Sciences of Araraquara, UNESP-Sao Paulo State University, Araraquara CEP 14801-902, Sao Paulo, Brazil; 2 Experimental Campus of Sao Vicente, UNESP-Sao Paulo State University, Sao Vicente CEP 11350-000, Sao Paulo, Brazil; 3 Department of Organic Chemistry, Institute of Chemistry, UNESP-Sao Paulo State University, Araraquara CEP 14800-900, Sao Paulo, Brazil

**Keywords:** mutagenicity, Ames test, flavonoids

## Abstract

The mutagenicity of ten flavonoids was assayed by the Ames test, in *Salmonella typhimurium* strains TA98, TA100 and TA102, with the aim of establishing hydroxylation pattern-mutagenicity relationship profiles. The compounds assessed were: quercetin, kaempferol, luteolin, fisetin, chrysin, galangin, flavone, 3-hydroxyflavone, 5-hydroxyflavone and 7-hydroxyflavone. In the Ames assay, quercetin acted directly and its mutagenicity increased with metabolic activation. In the presence of S9 mix, kaempferol and galangin were mutagenic in the TA98 strain and kaempferol showed signs of mutagenicity in the other strains. The absence of hydroxyl groups, as in flavone, only signs of mutagenicity were shown in strain TA102, after metabolization and, among monohydroxylated flavones (3-hydroxyflavone, 5-hydroxyflavone and 7-hydroxyflavone), the presence of hydroxyl groups only resulted in minor changes. Luteolin and fisetin also showed signs of mutagenicity in strain TA102. Finally, chrysin, which has only two hydroxy groups, at the 5-OH and 7-OH positions, also did not induce mutagenic activity in any of the bacterial strains used, under either activation condition. All the flavonoids were tested at concentrations varying from 2.6 to 30.7 nmol/plate for galangin and 12.1 to 225.0 nmol/plate for other flavonoids. In light of the above, it is necessary to clarify the conditions and the mechanisms that mediate the biological effects of flavonoids before treating them as therapeutical agents, since some compounds can be biotransformed into more genotoxic products; as is the case for galangin, kaempferol and quercetin.

## 1. Introduction

In recent years, there has been a growing academic and industrial interest in the health benefits of flavonoids [1]. Flavonoids are low-molecular-weight secondary metabolites of plants that, unlike primary metabolites, are not essential to their survival. Nevertheless, they are bioactive across kingdoms with over 9,000 structural variants known [2,3]. The diversity in size, three-dimensional shape and physical and biochemical properties of flavonoids allow them to interact with targets in a variety of subcellular locations, to influence biological activity in plants, animals, and microbes [4,5,6].

**Figure 1:**
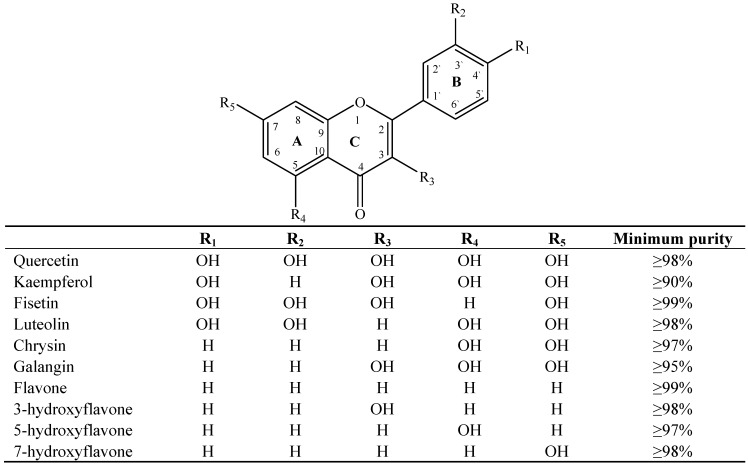
Molecular skeleton and purities (by HPLC) of flavonoids.

The basic molecular skeleton of flavonoid compounds (Figure 1) consists of two fused aromatic carbon rings, constituting benzopyran rings (A and C), and a benzene ring (B). These compounds can be divided into various sub-groups based on the degree of oxidation of the C-ring, the hydroxylation pattern of the ring structure and the substituent at position 3 [7], including chalcones, flavones, flavonols, flavanones, anthocyanins, and isoflavonoids [3].

One of the prominent and medically most useful properties of many flavonoids is their ability to scavenge free radicals [8], as well as anti-proliferative, antitumor, anti-inflammatory, and pro-apoptotic activities and some molecular targets have been identified. The health-promoting effects of flavonoids may relate to interactions with key enzymes, signaling cascades involving cytokines and transcription factors, or antioxidant systems [6].

Carcinogenicity and mutagenicity are among the toxicological effects that cause the highest concern for human health; thus, they are the object of intense research activity, as well as of recognized regulatory testing methods [9]. Generally, flavonoids have been characterized as antioxidants capable of providing beneficial health effects. However, many flavonoids have also been reported to be mutagenic in diverse strains of *Salmonella typhimurium *in the Ames test as well as in several mammalian cell systems used to assess different toxic end points [10]. In the field of flavonoid mutagenicity research, reports of activity are widely conflicting, probably owing to varying characteristics of chemical structure on inter- and intra-assay variation [11], and it seems that the activity is dependent either on the biotransformation of the compound or on the production of reactive oxygen species [12].

Thus, the objective of this study was to evaluate the mutagenic activity of the flavonoid compounds quercetin, kaempferol, luteolin, fisetin, galangin, chrysin, flavone, 3-hydroxyflavone, 5-hydroxyflavone, 7-hydroxyflavone (Figure 1) and their mutagenicity, measured by the *Salmonella *microsome assay (Ames test), in the presence and in the absence of *in vitro* metabolizing systems and investigate hydroxylation pattern-mutagenicity relationship profile. The mutagenic activities of compounds were assayed in the *S. typhimurium *tester strains TA98 (detects frameshift mutations), TA100 (detects base-pair-substitution mutations) and TA102 (normally used to detect mutagens that cause oxidative damage and base-pair-substitution mutations).

## 2. Results and Discussion

### 2.1. Results

Table 1 shows the mean number of revertants/plate (M), the standard deviation (SD) and the mutagenic index (MI) after the treatments with the various flavonoids, observed in *S. typhimurium *strain TA98, TA100 and TA102, in the presence (+S9) and absence (−S9) of metabolic activation.

In the absence of the external metabolizing system, S9 mix, quercetin is the only mutagenic flavonoid, with a mutagenic index higher than 2.0 at the concentration of 24.5 nmol/plate in strain TA98. Its mutagenicity in strain TA98 increases in the presence of metabolic activation in a dose-dependent manner, with a mutagenic index higher than 2.0 at 147.8 nmol/plate. In the TA100 strain, quercetin also induced an increase in the number of revertant colonies relative to the negative control in the absence of metabolic activation, with a mutagenic index of 2.0 at the concentration of 98.4 nmol/plate, also indicating the direct mutagenic activity for this strain. In the TA102 strain, no mutagenicity was detected in the absence of metabolic activation but in its presence, mutagenic effect was observed in strains TA100 and TA102 and the largest mutagenic indexes found were 3.2 and 2.1, respectively. According to the strains involved, quercetin, induces substitution of base pairs (TA100, TA102), oxidative damage (TA102) and, at a much higher rate, frameshift mutations (TA98).

Kaempferol also shows a dose-dependent induction of revertants in the TA98 strain and signs of mutagenicity in the other strains in the presence of S9 mix. Without metabolic activation, kaempferol does not induce any increase of revertants in this strain. The strains were rather sensitive to the toxic effects of galangin, and it was thus necessary to decrease the doses. In fact, the lowest dose used in the experiments with the other flavonoids was close to the highest dose used with galangin. The test concentrations of galangin varied from 2.6 to 30.7 nmol/plate and proved to be mutagenic only in the TA98 strain, in the presence of S9 mix. Flavone, luteolin and fisetin also showed signs of mutagenicity in the TA102 strain. None of the other flavonoids (chrysin, 3-hydroxyflavone, 5-hydroxyflavone and 7-hydroxyflavone) were mutagenic under the conditions used in this study. Table 2 summarizes the mutagenicity results.

### 2.2. Discussion

This work is based on the need to clarify the causes of the mutagenicity of 10 flavonoids, some of which have beneficial properties as antioxidants and anti-inflammatory activities and prevent cancer. Ames test was used which has been extensively employed as a screening tool to establish an initial estimate of mutagenicity and carcinogenicity [13].

Flavonoids are known to exhibit a variety of effects in different biological systems. They modulate the activities of enzymes involved in the biotransformation of precarcinogens, altering their biological activity, while under certain conditions flavonoids may exhibit genotoxic activity by yielding reactive intermediates, such as free radicals. It is therefore of importance that, depending on the assay performed, either *in vivo *or *in vitro*, a flavonoid may exhibit different properties in targets cells, depending on the concentration and its metabolic fate [14].

Early studies of flavonoids first documented the mutagenicity of quercetin and its capacity to cause base-pair substitutions and frameshift mutations in the Ames test [15], as well as to induce chromosomal aberrations and sister chromatid exchanges in CHO cells [16], but conflicting results were obtained in the micronucleus test *in vivo* [15,17,18]. The results obtained in this study indicate that there is a structure-activity relationship considering the pattern of hydroxyls presents in the molecule and depending on the tester strain.

According to Rietjens *et al*. [19], the structural features essential to mutagenic activity are a flavonoid ring structure with a free hydroxyl group at position 3, a double bond linking positions 2 and 3, and a keto-group at position 4, allowing the proton of the hydroxyl group at 3 to tautomerise to a 3-keto moiety. These structural features are exhibited by the molecule of quercetin, kaempferol, galangin, fisetin and 3-hydroxyflavone.

However, according to results obtained in this study, the hydroxylation pattern of quercetin was also important for the observed mutagenicity. Quercetin, which has two hydroxyl groups in the *ortho *position to each other on the B ring and free hydroxyl groups at position 3, 5 and 7 is directly mutagenic and its mutagenicity increases in the presence of metabolic activation. Quercetin showed greatest mutagenic activity in the *Salmonella *strain TA98, with mutagenic indices of 3.8 and 20.4, with and without S9 mix, respectively, at the highest concentration. In the *Salmonella* strains TA100 and TA 102, the mutagenic indices were 2.0 in the absence of metabolic activation in the TA100 and 3.2 and 2.1, respectively, after metabolization. The elimination or modification of either of these hydroxyl groups significantly reduced the mutagenicity, as observed in the results with kaempferol and galangin.

**Table 1:** Mutagenic activity expressed as the mean and standard deviation of the number of revertants/plate and the mutagenic index (MI), in bacterial strains TA98, TA100 and TA102 treated with flavonoids (quercetin, kaempferol, fisetin, luteolin, flavone, 3-hydroxyflavone, 5-hydroxyflavone, 7-hydroxyflavone, chrysin, galangin), at various doses, with (+S9) or without (−S9) metabolic activation.

	Treatments	Number of revertants∕plate in *S. typhimurium *strains (M ± SD) and (MI)					
		TA 98		TA 100		TA 102	
**Quercetin** 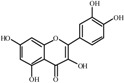	**nmol/plate**	**−S9**	**+S9**	**−S9**	**+S9**	**−S9**	**+S9**
	**0.0 ^a^**	53 ± 8	39 ± 1	115 ± 3.5	206 ± 5	273 ± 15	248 ± 3
	**12.1**	81 ± 0.6 * (1.5)	136 ± 5 ** (**3.5**)	134 ± 8.5 (1.2)	320 ± 2 * (1.5)	279 ± 8 (1.0)	305 ± 1 (1.2)
	**24.5**	115 ± 6 ** (**2.2**)	196 ± 4 ** (**5.0**)	178 ± 6.1 (1.5)	389 ± 4 ** (1.9)	302 ± 4 (1.1)	351 ± 13 (1.4)
	**49.1**	172 ± 1 ** (**3.3**)	393 ± 7 ** (**10.0**)	202 ± 3.2 ** (1.7)	492 ± 5 ** (**2.4**)	331 ± 6 (1.2)	446 ± 11 ** (1.8)
	**98.4**	135 ± 11 ** (**2.6**)	658 ± 10 ** (**16.9**)	227 ± 2.5 ** (2.0)	651 ± 4 ** (**3.2**)	324 ± 3 (1.2)	471 ± 1 ** (1.9)
	**147.8**	197 ± 4 ** (**3.8**)	794 ± 5 ** (**20.4**)	212 ± 5.0 ** (1.8)	642 ± 5 ** (**3.1**)	310 ± 6 (1.1)	524 ± 6 ** (**2.1**)
	**Ctrol+**	1347 ± 51 ^b^	1567 ± 47 ^e^	1582 ± 102 ^c^	1456 ± 67 ^e^	1656 ± 89 ^d^	1932 ± 93 ^f^
**Kaempferol** 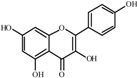	**0.0 ^a^**	58 ± 2	39 ± 1	106 ± 4	206 ± 53	235 ± 4	248 ± 3
	**14.3**	54 ± 5 (0.9)	141 ± 7** (**3.6**)	131 ± 1 (1.2)	286 ± 15 (1.4)	287 ± 4 (1.2)	336 ± 8 (1.3)
	**29.0**	56 ± 4 (1.0)	172 ± 3** (**4.4**)	146 ± 2 (1.4)	313 ± 5 (1.5)	258 ± 6 (1.1)	367 ± 7 (1.5)
	**58.0**	53 ± 1 (0.9)	199 ± 8** (**5.1**)	143 ± 4 (1.3)	369 ± 4 ** (1.8)	301 ± 9 (1.3)	430 ± 5 ** (1.7)
	**116.4**	58 ± 2 (1.0)	205 ± 3** (**5.3**)	130 ± 6 (1.2)	384 ± 12 ** (1.9)	305 ± 9 (1.3)	418 ± 8 (1.4)
	**174.7**	62 ± 3 (1.1)	177 ± 4** (**4.5**)	138 ± 3 (1.3)	400 ± 18 ** (1.9)	242 ± 9 (1.0)	473 ± 13 ** (1.9)
	**Ctrol+**	1324 ± 61 ^b^	1567 ± 47 ^e^	1457 ± 53 ^c^	1456 ± 67 ^e^	1473 ± 119 ^d^	1932 ± 93 ^f^
**Fisetin** 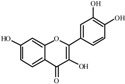	**0.0 ^a^**	50 ± 4	32 ± 1	146 ± 1	190 ± 1	264 ± 3	291 ± 8
	**14.3**	50 ± 1 (1.0)	41 ± 1 (1.3)	157 ± 7 (1.1)	209 ± 7 (1.1)	341 ± 11 (1.3)	333 ± 8 (1.1)
	**29.0**	52 ± 3 (1.0)	44 ± 2 (1.4)	142 ± 1 (1.0)	233 ± 6 (1.2)	334 ± 2 (1.3)	346 ± 10 (1.2)
	**58.0**	57 ± 1 (1.1)	40 ± 1 (1.2)	175 ± 6 (1.2)	232 ± 2 (1.2)	372 ± 4 (1.4)	348 ± 11 (1.2)
	**116.4**	58 ± 2 (1.1)	40 ± 2 (1.3)	133 ± 3 (0.9)	224 ± 8 (1.2)	472 ± 16 ** (1.8)	364 ± 15 (1.2)
	**174.7**	60 ± 1 (1.2)	36 ± 1 (1.1)	131 ± 3 (0.9)	232 ± 4 (1.2)	339 ± 15 (1.3)	387 ± 7 (1.3)
	**Ctrol+**	1425 ± 81 ^b^	1634 ± 88 ^e^	1325 ± 91 ^c^	1721 ± 72 ^e^	1689 ± 72 ^d^	1789 ± 90 ^f^
**Luteolin** 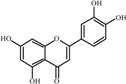	**0.0 ^a^**	49 ± 3	39 ± 1	141 ± 2	206 ± 5	265 ± 2	248 ± 3
	**14.3**	37 ± 2 (0.8)	33 ± 2 (0.8)	117 ± 3 (0.8)	226 ± 8 (1.1)	288 ± 6 (1.1)	264 ± 2 (1.1)
	**29.0**	45 ± 5 (0.9)	38 ± 3 (1.0)	129 ± 3 (0.9)	249 ± 10 (1.2)	282 ± 1 (1.1)	310 ± 7 (1.2)
	**58.0**	40 ± 3 (0.8)	35 ± 2 (0.9)	107 ± 3 (0.8)	253 ± 11 (1.2)	299 ± 16 (1.1)	380 ± 4 (1.5)
	**116.4**	45 ± 4 (0.9)	31 ± 3 (0.8)	150 ± 8 (1.1)	188 ± 5 (0.9)	340 ± 6 (1.3)	387 ± 6 * (1.6)
	**174.7**	48 ± 1 (1.0)	27 ± 1 (0.7)	148 ± 5 (1.0)	198 ± 13 (1.0)	288 ± 5 (1.1)	404 ± 3 * (1.6)
	**Ctrol+**	1376 ± 67 ^b^	1567 ± 47 ^e^	1276 ± 72 ^c^	1456 ± 67 ^e^	1767 ± 41 ^d^	1932 ± 93 ^f^
**Chrysin ** 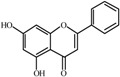	**0.0 ^a^**	49 ± 3	39 ± 1	156 ± 3	206 ± 5	235 ± 2	248 ± 3
	**16.1**	34 ± 5 (0.7)	34 ± 3 (0.9)	154 ± 2 (1.0)	222 ± 10 (1.1)	253 ± 4 (1.1)	305 ± 3 (1.2)
	**32.6**	40 ± 2 (0.8)	38 ± 2 (1.0)	141 ± 3 (0.9)	268 ± 7 (1.3)	298 ± 8 (1.3)	259 ± 2 (1.0)
	**65.3**	37 ± 2 (0.7)	33 ± 1 (0.8)	144 ± 2 (0.9)	274 ± 10 (1.3)	265 ± 5 (1.1)	233 ± 10 (0.9)
	**131.0**	44 ± 1 (0.9)	35 ± 3 (0.9)	151 ± 4 (1.0)	278 ± 15 (1.3)	282 ± 2 (1.2)	232 ± 6 (0.9)
	**196.7**	49 ± 1 (1.0)	32 ± 2 (0.8)	124 ± 5 (0.8)	254 ± 3 (1.2)	230 ± 7 (1.0)	231 ± 14 (0.9)
	**Ctrol+**	1376 ± 67 ^b^	1567 ± 47 ^e^	1579 ± 95 ^c^	1456 ± 67 ^e^	1377 ± 67 ^d^	1932 ± 93 ^f^
**Galangin ** 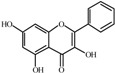	**0.0 ^a^**	22 ± 3	26 ± 1	208 ± 13	165 ± 1	144 ± 1	172 ± 5
	**2.6**	22 ± 2 (1.0)	34 ± 5 (1.3)	238 ± 2 (1.1)	209 ± 4 (1.3)	155 ± 4 (1.1)	144 ± 9 (0.8)
	**5.1**	23 ± 2 (1.0)	43 ± 2 * (1.6)	235 ± 3 (1.1)	207 ± 4 (1.3)	150 ± 4 (1.0)	162 ± 3 (0.9)
	**10.2**	23 ± 2 (1.0)	55 ± 3 ** (2.1)	233 ± 9 (1.1)	196 ± 5 (1.2)	148 ± 7 (1.0)	158 ± 6 (0.9)
	**20.5**	25 ± 4 (1.1)	57 ± 3 ** (2.1)	209 ± 9 (1.0)	177 ± 7 (1.1)	143 ± 1 (1.0)	151 ± 3 (0.9)
	**30.7**	26 ± 2 (1.2)	58 ± 3 ** (2.2)	199 ± 7 (1.0)	161 ± 9 (1.0)	127 ± 2 (0.9)	112 ± 7 (0.6)
	**Ctrol+**	1329 ± 66 ^b^	1422 ± 81 ^e^	1498 ± 45 ^c^	1667 ± 39 ^e^	1733 ± 77 ^d^	1980 ± 91 ^f^
**Flavone ** 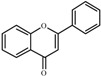	**0.0 ^a^**	35 ± 2	32 ± 1	173 ± 5	190 ± 1	313 ± 6	291 ± 8
	**18.4**	36 ± 2 (1.0)	38 ± 2 (1.2)	182 ± 2 (1.0)	221 ± 1 (1.2)	282 ± 3 (0.9)	350 ± 4 (1.2)
	**37.3**	34 ± 3 (1.0)	37 ± 2 (1.2)	174 ± 2 (1.0)	196 ± 4 (1.0)	251 ± 4 (0.8)	369 ± 7 (1.3)
	**74.7**	38 ± 1 (1.1)	36 ± 1 (1.1)	179 ± 1 (1.0)	208 ± 2 (1.1)	212 ± 7 (0.7)	414 ± 3 (1.4)
	**149.8**	35 ± 3 (1.0)	31 ± 2 (1.0)	144 ± 5 (0.8)	205 ± 4 (1.1)	136 ± 2 (0.4)	470 ± 8 * (1.6)
	**225.0**	46 ± 2 (1.3)	23 ± 2 (0.7)	106 ± 4 (0.6)	207 ± 5 (1.1)	109 ± 3 (0.3)	525 ± 6 ** (1.8)
3- **Hydroxyflavone** 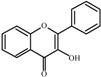	**0.0 ^a^**	50 ± 2	32 ± 1	194 ± 8	190 ± 1	235 ± 13	291 ± 8
	**17.2**	50 ± 4 (1.0)	39 ± 3 (1.2)	196 ± 6 (1.0)	235 ± 3 (1.2)	216 ± 11 (0.9)	308 ± 5 (1.1)
	**34.8**	43 ± 6 (0.8)	36 ± 3 (1.1)	207 ± 5 (1.1)	196 ± 2 (1.0)	207 ± 19 (0.8)	308 ± 7 (1.1)
	**69.7**	38 ± 7 (0.8)	34 ± 2 (1.1)	192 ± 5 (1.0)	192 ± 8 (1.0)	214 ± 8 (0.9)	334 ± 4 (1.1)
	**139.8**	48 ± 10 (0.9)	34 ± 2 (1.1)	217 ± 2 (1.1)	204 ± 6 (1.1)	212 ± 10 (0.9)	348 ± 3 (1.2)
	**209.9**	53 ± 1 (1.0)	27 ± 3 (0.8)	196 ± 5 (1.0)	212 ± 12 (1.1)	222 ± 16 (0.9)	348 ± 7 (1.2)
	**Ctrol+**	1337 ± 92 ^b^	1634 ± 88 ^e^	1523 ± 85 ^c^	1721 ± 72 ^e^	1384 ± 82 ^d^	1789 ± 90 ^f^
5- **Hydroxyflavone ** 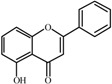	**0.0 ^a^**	32 ± 1	32 ± 1	177 ± 2	190 ± 1	306 ± 8	291 ± 8
	**17.2**	32 ± 2 (1.0)	26 ± 1 (0.8)	166 ± 3 (0.9)	202 ± 10 (1.1)	290 ± 7 (0.9)	353 ± 4 (1.2)
	**34.8**	36 ± 2 (1.1)	28 ± 2 (0.9)	156 ± (0.9)	208 ± 17 (1.1)	303 ± 5 (1.0)	361 ± 8 (1.2)
	**69.7**	30 ± 2 (0.9)	29 ± 3 (0.9)	185 ± 3 (1.0)	203 ± 6 (1.1)	296 ± 4 (1.0)	391 ± 8 (1.3)
	**139.8**	44 ± 2 (1.4)	31 ± 1 (1.0)	166 ± 3 (0.9)	206 ± 1 (1.1)	285 ± 4 (0.9)	405 ± 10 (1.4)
	**209.9**	34 ± 2 (1.1)	31 ± 4 (1.0)	142 ± 4 (0.8)	193 ± 8 (1.0)	265 ± 6 (0.9)	438 ± 6 (1.5)
	**Ctrol+**	1237 ± 61 ^b^	1634 ± 88 ^e^	1211 ± 72 ^c^	1721 ± 72 ^e^	1877 ± 52 ^d^	1789 ± 90 ^f^
7- **Hydroxyflavone** 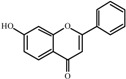	**0.0 ^a^**	50 ± 2	32 ± 1	136 ± 8	190 ± 1	229 ± 7	291 ± 8
	**17.2**	57 ± 4 (1.1)	30 ± 4 (0.9)	124 ± 3 (0.9)	211 ± 5 (1.1)	246 ± 25 (1.1)	344 ± 6 (1.2)
	**34.8**	56 ± 3 (1.1)	35 ± 3 (1.1)	121 ± 5 (0.9)	190 ± 9 (1.0)	270 ± 22 (1.2)	344 ± 5 (1.2)
	**69.7**	55 ± 8 (1.1)	36 ± 1 (1.1)	145 ± 6 (1.1)	205 ± 4 (1.1)	249 ± 13 (1.1)	345 ± 4 (1.2)
	**139.8**	65 ± 2 (1.3)	36 ± 1 (1.1)	143 ± 4 (1.0)	197 ± 8 (1.0)	246 ± 16 (1.1)	351 ± 13 (1.2)
	**209.9**	54 ± 4 (1.1)	39 ± 3 (1.2)	132 ± 18 (1.0)	177 ± 2 (0.9)	244 ± 16 (1.1)	369 ± 2 (1.3)
	**Ctrol+**	1337 ± 92 ^b^	1634 ± 88 ^e^	1398 ± 51 ^c^	1721 ± 72 ^e^	1277 ± 57 ^d^	1789 ± 90 ^f^

* *p* < 0.05 (ANOVA); ** *p* < 0.01 (ANOVA), M ± SD = mean and standard deviation; ^a^ Negative Control: dimethyl sulfoxide (DMSO—75 μL/plate); Positive Control (Ctrol+); ^b^ 4-nitro-*O*-phenylenediamine (10.0 μg/plate—TA98); ^c^ sodium azide (1.25 μg/plate—TA100); ^d^ mitomycin (0.5 μg/plate—TA102), in the absence of S9; and ^e^ 2-anthramine (1.25 μg/plate—TA98, TA100); ^f^ 2-aminofluorene (10.0 μg/plate—TA102), in the presence of S9. Values in brackets (MI) ≥2 indicate mutagenicity.

**Table 2:** Results of evaluation of mutagenicity of flavonoids.

Treatments	TA 98		TA 100		TA 102	
	−S9	+S9	−S9	+S9	−S9	+S9
Quercetin	+	+	+	+	-	+
Kaempferol	-	+	-	+∕-	-	+∕-
Fisetin	-	-	-	-	+∕-	-
Luteolin	-	-	-	-	-	+∕-
Chrysin	-	-	-	-	-	-
Galangin	-	+	-	-	-	-
Flavone	-	-	-	-	-	+∕-
3-Hydroxyflavone	-	-	-	-	-	-
5-Hydroxyflavone	-	-	-	-	-	-
7-Hydroxyflavone	-	-	-	-	-	-

(+) = positive; (-) = negative; (+∕-) = signs of mutagenicity.

The only structural difference between kaempferol and quercetin is the lack of the hydroxyl group at position 3' [20] and galangin is a flavonol that does not have any hydroxyl group on the B ring. In this study, kaempferol and galangin showed mutagenic activity in the *Salmonella* strain TA98 only after metabolic activation, with mutagenic indexes of 4.5 and 2.2, respectively, indicating that the number and position of hydroxyl groups on the B ring seem to have a specific influence on the genotoxicity of the compounds. Kaempferol also showed signs of mutagenicity in the other strains.

Galangin is a possible substrate of cytochrome P450 which, by hydroxylating the B ring, can metabolise it to kaempferol or even quercetin. This hypothesis has been raised by Brown and Dietrich [21]. Duarte Silva *et al*. [22] investigated if cytochrome P450 monooxygenase system is involved in the metabolism of galangin and consequently in its mutagenicity, and found that galangin really requires metabolic activation by cytochrome P450, in order to be mutagenic.

Kaempferol which has only one hydroxyl group on the B-ring exhibited a similar response to galangin. The mutagenicity of kaempferol in these cells also depends on its activation via the cytochrome P450 monooxygenase system [22]. The metabolization of kaempferol by the S9 mix gives rise to quercetin, increasing its genotoxicity [12].

The structural formula of quercetin contains all the structural elements required for both antioxidant and pro-oxidant activity [19] and these forms of quercetin may provide on insight into its apparent *in vitro* mutagenicity [23,24] or antimutagenicity.

A variety of genotoxicity assays have been conducted, because of the prevalence of quercetin in the diet and its potential clinical and food applications. Moreover, there is an on going attempt to reconcile the differences between *in vitro* results demonstrating quercetin-related mutagenic activity and the apparent absence of carcinogenicity *in vivo* [24].

In general, the molecule of quercetin becomes oxidized while exerting its antioxidative capacities and potentially toxic oxidation products are formed. *In vitro* studies define this effect as the quercetin paradox, *i.e*., the conversion of quercetin into a potential toxic product while offering protection by scavenging reactive oxygen species [24,25,26].

The mutagenicity mechanism of quercetin can or cannot involve metabolic activation, leading to the formation of catechol metabolites and subsequent oxidation to *o*-quinone and in some cases *p*-quinone methide metabolites. Quercetin already possesses a catechol group in the B-ring, which, due to its oxidant properties, provides a basis for the pro-oxidative toxic effects of this flavonoid [23]. These quinone methides are said to be alkylating DNA-reactive intermediates. An essential part of this hypothesis is that for mutagenic activity, the B-ring has to be oxidized to a quinoid intermediate and the proton of the hydroxyl group on C3 needs to rearrange to give a 3-keto isomer, which requires a double bond in the 2,3-position [12,19].

This explains why the absence of the hydroxyl on C3 eliminates the mutagenicity in the tested strains, as observed in the results with luteolin. Luteolin only showed signs of mutagenicity in strain TA102 after metabolization, with MI equal to 1.6.

However, in spite of the observation that the position of the 3-hydroxyl on the C ring is clearly important as indicated by the activity of galangin in the *Salmonella* strain TA98 after metabolic activation, the same effect was not observed for fisetin, which showed signs of mutagenicity in strain TA102 without metabolization (MI = 1.8).

Comparison of the mutagenicity of quercetin with that of fisetin also illustrates the potentiating effect of a hydroxyl group on C5. This effect occurs because this hydroxyl forms a strong hydrogen bond with the 4-keto group, thereby preventing and/or weakening the hydrogen bonding of the 3-hydroxyl with the 4-keto group. This weakening of the 3-hydroxyl 4-keto hydrogen bond increases the likelihood of tautomerisation of the 3-hydroxyl group to give the quinone methides [19]. Thus, the absence of the 5-hydroxyl (fisetin) also eliminates mutagenicity. But the presence of 5-hydroxy on the A ring is not also mutagenicity warranty, as observed in the results with 5-hydroxyflavone, that lacks mutagenicity, probably due the absence of other structural elements, as for instance 3-hydroxyl on the C ring.

The total absent of hydroxyl groups, as in flavone, induced signs of mutagenicity in strain TA102 after metabolization.

The presence of hydroxyl groups on the A ring, carbonyl and C2-C3 double bond on the C-ring seems not to be a determinant when considering the different flavone structures. Among monohydroxylated flavones (3-hydroxyflavone, 5-hydroxyflavone and 7-hydroxyflavone) the presence of hydroxyl groups results in only minor changes. Hydroxyl groups at carbons 3, 5 and 7 did not generate mutagenic compounds.

Finally, with chrysin, the two hydroxyl groups at positions 5 and 7 were not reactive with 4-keto group. Moreover the lack of the C-3 hydroxyl group and the 3',4' dihydroxy (or prenyl) on the B-ring of this flavonoid also can be the reason for the absent mutagenic activity.

## 3. Experimental

### 3.1. Chemicals and Culture Media

Quercetin, kaempferol, fisetin, luteolin, flavone, 3-hydroxyflavone, 5-hydroxyflavone, 7-hydroxyflavone, chrysin, galangin, dimethyl sulfoxide (DMSO), nicotinamide adenine dinucleotide phosphate sodium salt (NADP), D-glucose-6-phosphate disodium salt, magnesium chloride, L-histidine monohydrate, D-biotin, sodium azide, 2-anthramine and 2-aminofluorene were purchased from Sigma Chemical Co. (St. Louis, MO, USA). Oxoid Nutrient Broth No. 2 (Oxoid, Basingstoke, HAM, UK) and Bacto Agar (BD Bacto™, Sparks, MD, USA) were used as bacterial media. D-Glucose, magnesium sulfate, citric acid monohydrate, anhydrous dibasic potassium phosphate, sodium ammonium phosphate, monobasic sodium phosphate, dibasic sodium phosphate and sodium chloride were purchased from Merck (Whitehouse Station, NJ, USA).

### 3.2. Metabolic Activation System (S9 Mixture)

The S9 fraction, prepared from livers of Sprague-Dawley rats treated with the polychlorinated biphenyl mixture Aroclor 1254 (500 mg/kg), was purchased from Molecular Toxicology Inc. (Boone, NC, USA). The metabolic activation system consisted of 4% of S9 fraction, 1% of 0.4 M MgCl_2_, 1% of 1.65 M KCl, 0.5% of 1 M D-glucose-6-phosphate disodium and 4% of 0.1 M NADP, 50% of 0.2 M phosphate buffer and 39.5% sterile distilled water [27].

### 3.3. Salmonella Mutagenic Assay

Mutagenic activity was tested by the *Salmonella*/microsome assay, using the *Salmonella typhimurium* tester strains TA98, TA100 and TA102, kindly provided by B.N. Ames (Berkeley, CA, USA), with and without metabolization by the pre-incubation method [27]. The strains from frozen cultures were grown overnight for 12–14 h in Oxoid Nutrient Broth No. 2. The metabolic activation mixture (S9) was freshly prepared before each test. Five different doses of test compounds were assayed. All of them were diluted in DMSO. The concentrations were selected on the basis of a preliminary toxicity test. In all subsequent assays, the upper limit of the dose range tested was either the highest non-toxic dose or the lowest toxic dose determined in this preliminary assay. Toxicity was apparent either as a reduction in the number of His*+* revertants, or as an alteration in the auxotrophic background (*i.e*., background lawn). The concentrations varied from 2.6 to 30.7 nmol/plate for galangin and 12.1 to 225.0 nmol/plate for the other flavonoids. The various concentrations of compounds to be tested were added to 0.5 mL of 0.2 M phosphate buffer (pH 7.4) or with 0.5 mL of 4% S9 mixture and 0.1 mL of bacterial culture and then incubated at 37 °C for 20–30 min. After this time, 2 mL of top agar was added to the mixture and poured on to a plate containing minimal agar. The plates were incubated at 37 °C for 48 h and the revertant colonies were counted manually. All experiments were analyzed in triplicate. The results were analyzed with the Salanal statistical software package (U.S. Environmental Protection Agency, Monitoring Systems Laboratory, Las Vegas, NV, version 1.0, from Research Triangle Institute, RTP, North Carolina, USA) [28], adopting the Bernstein *et al*. [29] model. The data (revertants/plate) were assessed by analysis of variance (ANOVA), followed by linear regression. The mutagenic index (MI) was also calculated for each concentration tested, this being the average number of revertants per plate with the test compound divided by the average number of revertants per plate with the negative (solvent) control. A sample was considered mutagenic when a dose-response relationship was detected and a two-fold increase in the number of mutants (MI ≥ 2) was observed with at least one concentration [30]. When only one of these criteria was met, the sample was considered to present signs of mutagenicity, in agreement with McGeorge *et al.* [31]. The standard mutagens used as positive controls in experiments without S9 mix were 4-nitro-*O*-phenylenediamine (10 µg/plate) for TA98, sodium azide (1.25 µg/plate) for TA100 and mitomycin (0.5 µg/plate) for TA102. 2-Anthramine (1.25 μg/plate) was used with TA98 and TA100 and 2-aminofluorene (1.25 μg/plate) with TA102 in the experiments with metabolic activation. DMSO served as the negative (solvent) control (75 µL/plate).

## 4. Conclusions

Given the above results and hypotheses, it is still necessary to clarify the mechanisms and the conditions that mediate the biological effects of flavonoids, before considering them as therapeutic agents. It is true that flavonoids may inhibit the bioactivation of precarcinogens by repressing several biotransformation enzymes or by blocking membrane receptors involved in the cellular uptake of precarcinogens. However, these compounds may themselves be biotransformed into more genotoxic products, as is the case for galangin, kaempferol and quercetin.
